# A Primer on Detecting Cirrhosis and Caring for These Patients without Causing Harm

**DOI:** 10.4061/2011/801983

**Published:** 2011-10-26

**Authors:** Bruce Allen Runyon

**Affiliations:** Department of Internal Medicine, Division of Gastroenterology/Hepatology, Loma Linda University Medical Center, 11234 Anderson Street, Room 1556, Loma Linda, CA 92354, USA

## Abstract

Many people who have cirrhosis are undiagnosed. The diagnosis may not become evident until they develop multiorgan failure after an invasive procedure. Patients with cirrhosis are unusually fragile and can be easily harmed and even set into a fatal down-spiral by seemingly innocuous treatments including medications and invasive procedures. There is much confusion regarding the care of these patients. For example, what medications can be used safely to treat pain, what sedatives are safe and effective, which medications are to be avoided, what diet should be prescribed, and which invasive procedures are safe. This paper provides the author's advice regarding clues to the presence of cirrhosis and the dos and do nots in the general care of these patients, based on his 30 years of experience in a liver-failure-focused academic practice.

## 1. Introduction

Cirrhosis is increasing in prevalence due to the epidemics of hepatitis C and obesity and the associated fatty liver [1]. Alcohol use/abuse is increasing in many countries, in part due to the global recession [2]. Many patients with cirrhosis at this time have two or even all three of the above insults speeding liver damage and scarring. It is estimated that there may be 10,000,000 people with cirrhosis in the US, ~3% of the population. Many if not most of these patients are not diagnosed yet with cirrhosis. Many exhibit signs or clues to the presence of cirrhosis (Table 1). Physicians should be alert to these signs and clues and then take the necessary steps to evaluate them or refer them for evaluation and avoid causing harm. 

## 2. Clues to the Presence of Cirrhosis

Some clues for the primary care provider or the specialist that cirrhosis may be present can be found in a careful history, physical examination, routine laboratory testing, and imaging. The author is amazed how commonly these useful clues are ignored. 

Family medicine physicians and general internists should be suspicious of the presence of cirrhosis in their patients with metabolic syndrome and/or long-standing diabetes [3]. Many of these patients will have silent cirrhosis due to nonalcoholic steatohepatitis (NASH). 

Many people who abuse alcohol and/or drugs also smoke. Pulmonologists and ear, nose, and throat specialists should be alert to the possibility of cirrhosis in their patients with chronic lung disease, lung cancer, or head and neck cancer. Too often invasive, curative procedures are performed to treat cancer, without recognition that cirrhosis is present; and the patient dies tumor-free, but is dead nonetheless. 

Preoperative assessment of the risks versus benefits of an invasive procedure is much more accurate once the presence of cirrhosis is known or suspected. Referral of the patient to a hepatologist for pre-operative “clearance” is wise. A less invasive procedure may be recommended. The patient may be more likely to survive a less invasive procedure rather than the state-of-the-art aggressive procedure.

## 3. History

Risk factors for cirrhosis include long-standing obesity (especially if insulin resistance or overt diabetes is present), chronic alcohol use (>14 drinks/week for women and >28 drinks/week for men), transfusion prior to 1992, and street drug use [4]. Many patients who have shared needles in the use of drugs, with resulting hepatitis C, are reluctant to divulge this information but may admit to cocaine snorting or other drug use. Tattoos and acupuncture can transmit hepatitis C or B, although the risk is not very high. Birth in Southeast Asia, areas in South America, or Africa is a risk factor for hepatitis B. Hepatitis C is hyperendemic in Italy, Egypt, other parts of the Middle East, Vietnam, and Japan. Children who have lived in chronic care facilities are at risk for hepatitis B due to biting and other activities that occur in these institutions. Sexual promiscuity, especially in men who have sex with men, is high risk for hepatitis B [4]. 

Sleep/wake reversal commonly precedes overt hepatic encephalopathy (HE). Standard medications for insomnia regularly unmask the tendency for confusion and can bring on frank coma. 

A history of abdominal enlargement and/or edema, episodic confusion, or bleeding from the gut should all raise strong suspicion of cirrhosis.

## 4. Physical Examination

Palmar erythema, vascular spiders, and abdominal collaterals are useful to look for on physical examination. Their presence should raise suspicion of cirrhosis. Parotid enlargement is more common in alcohol abusers than in patients with nonalcoholic liver disease and is not specific for liver disease. A firm liver or palpable spleen also raises suspicion of cirrhosis. A past president of the American Association for the Study of Liver Diseases used to say: “one good feel of the liver is better than any two liver tests.” This is a very insightful statement. 

The presence of jaundice, ascites (with or without edema), or asterixis provides evidence of advanced liver injury.

## 5. Laboratory Testing

Eugene Schiff tells us that he “has great respect for the platelet count” as a clue for the presence of cirrhosis; this is also a very insightful statement. Almost everyone has a platelet count in their medical database whether liver disease is suspected or not. A platelet count <160,000 × 10(9)/L is 80% sensitive in detecting portal hypertension from cirrhosis in patients with chronic hepatitis C [5]. The white blood cell count may also be below the normal range, but this is less common than the thrombocytopenia of cirrhosis. Anemia (~6.5 mmoL/L) may be present when ascites develops, but hemoglobin may be normal prior to decompensation. 

Any patient with a platelet count <160,000 × 10(9)/L should be suspected of having cirrhosis, especially if there is other evidence of liver disease in the history, physical examination, or laboratory testing. 

Many anesthesiologists will postpone/cancel an invasive procedure when the platelet count is below the normal range. The optimal normal range can be debated; however, a value below 160,000 is a very useful parameter in this author's experience—to cancel the procedure and request a hepatology consult. It is also this author's experience that in relatively healthy outpatients, thrombocytopenia is more common in the setting of liver disease than it is in primary hematologic conditions. Although hematology consultation is common in these patients, it is usually unnecessary and simply delays evaluation by the hepatologist. 

Fatty liver is extremely common now in the US and in many other developed countries and is the usual explanation for an abnormal aspartate aminotransferase (AST) or alanine aminotransferase (ALT) [6]. An amazing 58% of Hispanics, 44% of Whites, and 35% of African Americans had fatty liver by biopsy after initial ultrasound detection of steatosis in an army medical clinic in Texas [7]. A variable percent of these patients have abnormal aminotransferases. Approximately 20% of patients with fatty liver will develop cirrhosis in 20 years time [8]. The usual pattern of aminotransferases in precirrhotic fatty liver is ALT > AST. Once cirrhosis develops, this pattern may reverse. 

When the alkaline phosphatase and/or bilirubin are also elevated, liver disease is essentially assured and an evaluation of the cause and severity is in order. 

Blood urea nitrogen is regularly below the normal range in patients with cirrhosis, and the international normalized ratio (INR) is regularly increased. The INR, creatinine, and bilirubin are the components of the model for end-stage liver disease (MELD) score, which quantifies severity of cirrhosis and prognosis [9].

## 6. Imaging

Generalists tend to order computed tomographic (CT) scans in pursuit of abdominal pain or other abdominal pathology. Hepatologists tend to perform or order ultrasounds initially and then order a CT scan once cirrhosis is suspected on ultrasound. CT appears much more sensitive than ultrasound in detecting hepatocellular carcinoma. 

Imaging without mention of a specific maximum spleen dimension is not very useful. This author usually orders an “ultrasound of the liver and spleen with Doppler.” If the spleen dimension is not included in the report, this author asks the radiologist to provide this crucial information. Although many radiologists use 13 cm as the threshold for splenomegaly, this author has seen many patients, with cirrhosis and portal hypertension, whose spleen was between 12 and 13 cm. It is this author's opinion that 12 cm should be the threshold to diagnose splenomegaly and raise suspicion of cirrhosis with portal hypertension. 

Imaging may also demonstrate nodularity, atrophy of the entire liver, atrophic right lobe, hypertrophied left lobe, a dilated portal vein (>13 mm), collaterals, and spontaneous splenorenal shunt—findings all consistent with the presence of cirrhosis [10]. 

When ascites is present in association with signs of cirrhosis and portal hypertension, advanced liver disease is essentially assured. Gallbladder and bowel wall edema are common when ascites is present, due simply to abdominal edema; these finding do not imply the presence of significant gallbladder or bowel pathology. 

Due to these patients' frequent risk factors for cancer, they may have malignancy-related ascites contributing to fluid retention in addition to cirrhosis [11]. 

Some patients with cirrhosis do not know that they have it and do not seek medical attention until they have developed a complication of advanced hepatocellular carcinoma, such as malignant portal vein thrombosis. This in turn may be manifested by new-onset ascites ot variceal hemorrhage. Ascites and/or variceal hemorrhage can be the clinical complications that lead to the request for the CT or ultrasound. 

Although magnetic resonance imaging may be the most sensitive modality in detecting hepatocellular carcinomas, it may be too sensitive in detecting dysplastic nodules and may lead to more confusion than clarity [12]. Local equipment, expertise on the part of the radiologist, and patient cooperation with breath holding all impact image quality. Also it may be difficult to obtain due to its high cost compared to other modalities. In general this author reserves this modality to further clarify focal lesions that are detected on ultrasound or CT.

## 7. Sensitivity to Coumadin

Patients with cirrhosis can be unusually sensitive to the anticoagulant effects of coumadin. This author has seen a patient who was supratherapeutic with a subdural hematoma taking only 0.5 mg of coumadin fives days per week. Patients who are therapeutic or supratherapeutic on unusually low doses of coumadin should be suspected of having cirrhosis.

## 8. Referral

If there is evidence of cirrhosis based on history, physical examination, laboratory testing, or imaging, referral of the patient to a hepatologist or liver-focused gastroenterologist may be in order. These subspecialists are most adept in prioritizing the many problems of the patients with cirrhosis, and in treating those problems, with the lowest risk of harming the patient in the process.

## 9. Liver Biopsy

Most patients with cirrhosis do not need a liver biopsy. The causes(s) and severity are usually evident without the need for histologic examination. Risks must be weighed against benefits. The biopsy is performed only if it will have an impact on treatment. Transjugular biopsy with pressures is preferable when ascites and/or coagulopathy are present. 

## 10. Liver Transplantation

Liver transplantation can be considered, based on psychosocial issues, severity of liver failure, and comorbidities. Early referral is preferable to late referral in order to assure insurance coverage for transplant, reduction in comorbidities, change in life style, and screening for treatable disease. An elective evaluation is preferable to an emergent evaluation after the patient sustains a major deterioration. 

## 11. General Advice to Patients

Many patients with cirrhosis have multiple insults to the liver including alcohol use/abuse. Abstinence from alcohol can dramatically improve their overall condition, even in the continued presence of chronic hepatitis C and/or obesity [13] (Table 2). Many patients with alcoholic liver disease can have alcoholic hepatitis superimposed on cirrhosis [14]. Patients who have a major component of alcoholic hepatitis can recompensate dramatically with abstinence. Ascites that has been refractory to diuretics can become diuretic responsive again and can even disappear such that diuretics can be discontinued. Chronic hepatitis B with decompensation and treatment with an antiviral is essentially the only other form of liver failure in which this sequence can occur. 

Abstinence is key to survival of the patient with decompensated alcoholic liver disease; those who continue to drink are all dead within three years [15]. In stark contrast, approximately 3/4 of those who totally abstain can survive three years [15]. 

Baclofen, 5 mg orally thrice daily for three days, then 10 mg orally thrice daily for a total of 90 days, has been shown in a carefully performed randomized, double-blind, controlled trial to dramatically decrease alcohol craving and alcohol use, specifically in patients with alcoholic liver disease [16]. Prior trials of drugs to reduce alcohol use specifically excluded patients with liver disease. This author has had very good success with this drug in safely reducing and actually eliminating alcohol consumption with treatment duration greater than twelve months [14, 17]. 

## 12. Diet

Diet is a cause of confusion and controversy in cirrhosis. Obesity speeds hepatitis C liver injury, speeds alcoholic liver injury and can cause cirrhosis in the absence of other insults [8, 18]. Obesity is best prevented. Once it is present and the patient has developed cirrhosis, the fat tends to disappear from the liver and patients regularly spontaneously lose weight [8]. At this late stage it may be too late for further weight reduction to be beneficial to the liver. We need prospectively collected data regarding this topic. 

It is reasonable to advise patients with risk factors for cirrhosis to maintain their body-mass index <25 or at least <30 to minimize the risk of fatty liver contributing to further liver damage. 

“Prophylactic” sodium or protein restriction is not data supported and could aggravate or induce malnutrition. Sodium restriction should only be advised once fluid retention has developed. A daily 2000 mg sodium restriction is the most data supported and can be followed without purchase of special foods [19]. Fluid restriction is not necessary unless the serum sodium drops below 120 mmol/L; even then its utility is not data supported [19]. Protein restriction is almost never needed—only when a patient has extremely severe hepatic encephalopathy that is unresponsive to two drugs and restriction of meat protein. This only occurs after surgical, interventional radiologic, or spontaneous portacaval shunts, in this author's experience. 

## 13. Raw Oysters and Clams

This author gives his patients with cirrhosis written instructions not to eat raw shellfish. Establishments in California that sell raw shellfish must display a visible sign warning patients with liver disease that these foods can cause a serious and potentially fatal infection. This infection is due to *Vibrio vulnificus*. Patients with cirrhosis are unusually vulnerable to this infection [20]. This author has seen only one survivor who had to have both legs amputated.

## 14. Herbals

Many people are taking herbals or food supplements for health maintenance or for self-treatment of disease. This practice can cause harm to the liver since some of these agents are hepatotoxic. These substances are not regulated by the Food and Drug Administration (FDA), may not be pure, and may contain no actual active ingredient. One of the few data-supported agents that may benefit patients with cirrhosis is vitamin D. Patients with cirrhosis are usually deficient in vitamin D; deficiency correlates with worse disease severity [21, 22]. Measurement of the vitamin D level and supplementation to achieve a normal level may help in many ways to improve the health of these patients. 

It is possible that milk thistle can benefit patients with hepatitis C or fatty liver. The ongoing National Institutes of Health trial will eventually answer this question. If this trial is positive, this drug should be FDA approved, available in a pure, regulated form, and insurance should pay for it. 

## 15. Heavy Lifting

When a patient bears down and does a Valsalva maneuver to pick up something heavy, he/she can dramatically increase the pressure in esophageal varices. These varices can suddenly bleed. This author has seen this happen many times. This author advises patients with esophageal varices to limit the weight that they lift to 40 pounds (18 kilograms). 

## 16. Nuisance Symptoms

Many patients with cirrhosis have insomnia, muscle cramps, nausea, pruritus, unintentional weight loss, and meralgia paresthetica. Patients may not spontaneously complain of these symptoms but regularly confirm their presence when asked. 

## 17. Sedatives

Many physicians prescribe standard sedatives for insomnia with or without knowing that the patient has cirrhosis. Sedatives can unmask the patient's tendency for hepatic encephalopathy and can even cause frank coma. It is useful to order a urine drug screen when a patient with evidence of cirrhosis is in the emergency department with mental status change. Prescription sedatives (which may or may not belong to the patient) or sedating street drugs may be detected. Discontinuing these agents can solve the patient's confusion problem. 

The only drug that has been shown to be effective specifically for insomnia of cirrhosis is hydroxyzine at 25 mg at night [23]. This author has been using trazodone 100 mg at night for patients with cirrhosis and insomnia; this drug can help with insomnia and can also treat depression that may be overt or covert in these patients. 

## 18. Muscle Cramps

Muscle cramps are common is patients with cirrhosis, especially once fluid retention develops. Many patients have muscle cramps despite normokalemia. Quinidine has been shown to be effective in a randomized trial [24]; however, the risk of sudden death from quinidine probably outweighs the benefit of this drug. Quinine sulfate works well in this author's experience but is no longer available over the counter and is not FDA approved for muscle cramps any longer. Patients can get quinine sulfate in tonic water. This author advises them to drink a glass or two per day and then continue this as needed to prevent the cramps. 

## 19. Nausea

Nausea of liver disease, especially the patient with deep jaundice, is a nuisance symptom that was well controlled with cisapride. However, this drug is essentially not available any longer either. Metoclopramide can be helpful in controlling nausea and seems more effective than ondansetron in patients with liver disease.

## 20. Pruritus

Pruritus is most characteristic of primary biliary cirrhosis but can be present in any patient with jaundice and even in anicteric patients with hepatitis C. Cholestyramine is the most effective treatment; however, patients do not like to take it, and there are drug/drug interactions. It should be taken 1 hour before or 2 hours after other drugs to maximize absorption of those drugs. Sertraline has been shown to decrease pruritus in primary biliary cirrhosis in a randomized trial [25]. 

## 21. Muscle Weight Loss

Many patients with cirrhosis lose body weight including muscle weight when they decompensate. Two cans of nutritional supplement after 9 pm have been shown to increase muscle mass in cirrhosis in a randomized trial [26]. 

## 22. Meralgia Paresthetica

Numbness and tingling of the lateral thigh can be due to injury to the lateral femoral cutaneous nerve, due to pressure induced by ascites. This meralgia paresthetica is a cause of concern among patients and a cause of confusion among physicians. It can improve with control of fluid overload and does not warrant specific treatment otherwise [27].

## 23. Screening and Prevention

Patients with suspected or proven cirrhosis should be screened for causes of cirrhosis and for complications of cirrhosis. Preventative measures and treatments should be provided when appropriate.

**Body Mass Index (BMI)**

As mentioned previously many patients with cirrhosis have more than one liver insult. This author has a new patient consult preprinted form that includes details such as number of years of BMI >25, number of years of BMI >30, and number of years of diabetes or prediabetes. We measure the patient's height and weight. This author has given up asking about height. Patients are embarrassed when their measured height is essentially always shorter than their stated height—even six inches! These details provide some clues regarding the possibility of NASH playing a role in causing cirrhosis.

## 24. Alcohol

This author also asks patients when they first started drinking, what they drink, how many drinks daily for how many years, as well as how many public intoxication charges and how many arrests for driving under the influence of alcohol they have had. These details provide evidence regarding the role of alcohol in causing cirrhosis.

## 25. Risk Factors for Chronic Hepatitis

This author asks about risk factors for chronic hepatitis, including intravenous drug use (even once), cocaine use, transfusion prior to 1992, dialysis, birth in a country where hepatitis B is endemic, homosexual behavior, carrier in the family, tattoos, and acupuncture. 

Blood should be tested for hepatitis C antibody, total antibody for hepatitis A, and three tests for hepatitis B, including surface antigen, total core antibody, and surface antibody. Those who are not immune to hepatitis A and B should be vaccinated for those viruses to prevent acute on chronic liver failure. 

## 26. Other Serologic Testing

Testing for autoantibodies, iron, ceruloplasmin, and alpha-1 antitrypsin phenotype should be performed, depending on the age of the patient and other risk factors. 

## 27. Varices and Hepatocellular Carcinoma

All patients with cirrhosis should be considered for screening for esophageal varices by upper endoscopy and for hepatocellular carcinoma. This author used to avoid screening very elderly patients, that is, those >80 years of age. However, variceal hemorrhage can occur in this age group. Hemorrhage is better prevented through endoscopy and beta blockade rather than being treated after it occurs. When large varices are detected in patients who have not bled, a beta blocker should be prescribed; survival is improved by this strategy [28]. The details of screening for hepatocellular carcinoma are somewhat controversial. The practice guideline recommends ultrasound twice yearly [29]. This author obtains an ultrasound once yearly, a CT once yearly, and alpha fetoprotein twice yearly. Ultrasound is a very insensitive modality in detecting this tumor; CT is preferable but involves radiation and potential nephrotoxicity [12]. This author obtains one of these modalities every six months. 

## 28. Drugs in Patients with Cirrhosis

### 28.1. Pain Control

Pain control is a major issue in patients with cirrhosis. Narcotic use decreases pain threshold; many prior intravenous narcotic users switch to prescription narcotics and become “drug seekers,” asking for narcotics from every physician who they encounter. The “5th vital sign” is a huge problem in this regard. Forcing physicians by law to prescribe narcotics when patients claim to have severe pain has been associated with a dramatic increase in narcotic use as well as fatal and nonfatal narcotic overdoses [30]. This author opposes use of narcotics in patients with cirrhosis unless they have cancer-related pain. The physician who prescribes narcotics liberally can become an “enabler” of the patient's addiction. 

Narcotics regularly unmask the tendency to HE and aggravate HE. Stopping the narcotic use can eliminate HE or make it easier to treat. Patients with cirrhosis on methadone can develop HE as their cirrhosis worsens; tapering the methadone dose to zero can help with treatment of their confusion. 

Similarly other drugs that cause constipation can aggravate HE and should be stopped when HE becomes a problem. 

### 28.2. NSAIDs

Nonsteroidal anti-inflammatory drugs (NSAIDs) are poorly tolerated by patients with cirrhosis. Approximately 1/3 of patients with cirrhosis who bleed from the upper gut have taken an NSAID within two weeks of the hemorrhage [31]. This author gives patients with cirrhosis written instruction not to take a single NSAID pill as long as they live. A rare patient whose coronary artery disease exceeds the severity of his cirrhosis may be permitted to take an 81 mg aspirin daily. 

### 28.3. Acetaminophen

Many physicians are confused about pain control in cirrhosis. Some actually prohibit acetaminophen use and recommend NSAIDs instead. This strategy is simply wrong and regularly harms the patient. Acetaminophen is tolerated better by patients with cirrhosis than by normal healthy people [32]. It is the active metabolite that is hepatotoxic. This author has been advising patients in writing for two decades that 4000 mg daily of acetaminophen is acceptable. There are no data to support a maximum dose of 2000 mg daily [32]. That dose does not control continuous pain. If pain is not controlled with 4000 mg daily, this author adds tramadol at 50 mg thrice daily and occasionally 100 mg thrice daily for a large patient with severe pain. This author has not seen problems with these doses of these drugs in patients with cirrhosis. 

### 28.4. Hepatotoxins and Nephrotoxins

The risks versus benefits of every medication must be carefully considered in patients with cirrhosis. Although patients with cirrhosis do not have a higher statistical chance of hepatotoxicity from drugs, they have less “reserve” than people with normal livers and may develop acute-on-chronic liver failure. Isoniazid is a notorious example of this phenomenon. Patients with cirrhosis should be considered for nonisoniazid-based antituberculosis treatment and the threshold for treating “gray-zone” patients should be high. Many patients with cirrhosis are from countries where Bacillus Calmette-Guerin vaccinations are given. They may have a false-positive skin test for tuberculosis. Isoniazid-associated fatal hepatotoxicity is reported in such patients, even in the absence of cirrhosis [33]. 

Patients with cirrhosis and azotemia may not tolerate intravenous contrast. If the serum creatinine is >1.5 or estimated clearance is <30 mL/min, it may be prudent to avoid the contrast. Aminoglycosides are also poorly tolerated by patients with cirrhosis and should be avoided unless there is no other option.

## 29. General Philosophy of Drug Use in Cirrhosis

One of the first things that this author does when evaluating a new consult patient is to remove most of the drugs from their list. The fewer drugs, the better. Complicated drug regimens are regularly abandoned by these patients. This author's philosophy is to prescribe only the crucial drugs and stop the others. Some funding sources only provide six drugs per month. Prescribing useless/borderline efficacy drugs may prevent these patients from receiving crucial drugs that keep them compensated and out of the hospital, such as diuretics. 

Vitamins other than vitamin D are not data supported for long-term use in patients who are eating well and do not have malabsorption. Alcoholics usually receive folate and thiamine when hospitalized. However, there is no evidence that long-term continuation of these drugs is useful. Vitamin K is given parenterally (intravenously or subcutaneously but not intramuscularly) to hospitalized patients with coagulopathy. Oral vitamin K is not well absorbed and is one of the useless drugs mentioned above. 

## 30. Invasive Procedures

Minor procedures such as abdominal paracentesis are well tolerated by patients with cirrhosis even without prophylactic transfusions of plasma or platelets [19]. There is no data-supported cutoff beyond which these procedures cannot be done. The risks versus benefits of each procedure must be carefully considered. The benefits of abdominal paracentesis almost always outweigh the risks

## 31. Surgery

Patients with cirrhosis regularly die due to multi-organ failure after abdominal or thoracic surgical procedures [34, 35]. Cranial and orthopedic surgery may be better tolerated [34]. Postoperative mortality can be estimated by “MELD 9,” http://www.mayoclinic.org/meld/mayomodel9.html. Too often the presence of cirrhosis was not detected preoperatively. It may be detected intraoperatively when a nodular liver and/or large abdominal collateral are seen, or postoperatively when jaundice and multi-organ failure develop. 

Referring physicians as well as surgeons and anesthesiologists regularly ignore the clues and signs that should have been acknowledged and should have led to postponement of the procedure and hepatology consultation for preoperative clearance. 

When this author is asked to “clear” patients for surgery, he prints out the MELD 9 estimate of 7-day, 30-day, 90-day, 1-year and 5-year mortality. He discusses these estimates with the patient and their family and usually recommends a minimally invasive procedure instead of the state-of-the-art maximally invasive procedure. For example, radiation or laser therapy or fulguration can be given instead of a resection. The prognosis of many patients with cirrhosis is worse than their prognosis from the untreated tumor. Symptoms caused by the tumor and life expectancy from both conditions must be weighed. This author has seen patients undergo fatal resection of a previously treated (radiation and chemotherapy) colon cancer; there was no residual malignancy in the resected specimen. This is a tragedy that is preventable by careful evaluation of the risks versus benefits of surgery and consideration of the possibility that the tumor is already cured.

## 32. Conclusions

Many people who have cirrhosis remain undiagnosed. Careful attention to risk factors for liver disease and clues to the presence of cirrhosis on history, physical examination, laboratory testing, and imaging can rule in the presence of cirrhosis and help avoid harmful treatments. Cirrhosis has an impact on medication usage and mortality after invasive procedures. The fewer medications taken by these patients, the better their outcome. The fewer invasive procedures performed on these patients, the better the outcome. The risks versus benefits of medication and each procedure must be carefully weighed. Do no harm is the relevant dictum.

## Figures and Tables

**Table 1:** Clues to the presence of cirrhosis.

Risk factors for liver disease, especially obesity, alcohol use, and high-risk behavior for hepatitis C
Sleep/wake reversal
History of fluid retention, gastrointestinal hemorrhage, or unexplained episodic confusion
Stigmata of cirrhosis, including palmar erythema, vascular spiders, and abdominal wall collaterals
Firm liver and/or enlarged spleen
Jaundice, ascites, or asterixis
Platelet count <160,000 × 10(9)/L
Abnormal liver tests
Prolonged international normalized ratio (INR)
Evidence of cirrhosis and/or portal hypertension on imaging
Unusual sensitivity to coumadin

**Table 2:** General advice regarding the care of patients with cirrhosis.

Total abstinence from alcohol
Consider baclofen to reduce/eliminate alcohol craving and alcohol use
Minimize medications, herbals, and food supplements
Avoid overweight/obesity
No prophylactic diets
Avoid raw oysters and clams
Consider vitamin D
Avoid lifting >40 pounds (18 kilograms)
Treat nuisance symptoms as needed
Screen for treatable components to liver injury, varices, and hepatocellular carcinoma
Avoid sedatives, narcotics, and tranquilizers
Use acetaminophen and tramadol and avoid nonsteroidal anti-inflammatory drugs
Avoid hepatotoxins and nephrotoxins
Carefully weigh risks versus benefits of all invasive procedures, especially surgery
Use MELD 9 to estimate operative mortality
Do no harm

## References

[1] Russo MW, Wei JT, Thiny MT (2004). Digestive and liver diseases statistics, 2004. *Gastroenterology*.

[2] Burki T (2010). Changing drinking patterns: a sobering thought. *The Lancet*.

[3] Marchesini G, Bugianesi E, Forlani G (2003). Nonalcoholic fatty liver, steatohepatitis, and the metabolic syndrome. *Hepatology*.

[4] Mitchell AE, Colvin HM, Beasley RP (2010). Institute of medicine recommendations for the prevention and control of hepatitis B and C. *Hepatology*.

[5] Pilette C, Oberti F, Aubé C (1999). Non-invasive diagnosis of esophageal varices in chronic liver diseases. *Journal of Hepatology*.

[6] Clark JM, Brancati FL, Diehl AM (2003). The prevalence and etiology of elevated aminotransferase levels in the United States. *The American Journal of Gastroenterology*.

[7] Williams CD, Stengel J, Asike MI (2011). Prevalence of nonalcoholic fatty liver disease and nonalcoholic steatohepatitis among a largely middle-aged population utilizing ultrasound and liver biopsy: a prospective study. *Gastroenterology*.

[8] Powell EE, Cooksley WGE, Hanson R, Searle J, Halliday JW, Powell LW (1990). The natural history of nonalcoholic steatohepatitis: a follow-up study of forty-two patients for up to 21 years. *Hepatology*.

[9] Kamath PS, Kim WR (2007). The model for end-stage liver disease (MELD). *Hepatology*.

[10] Aubé C, Oberti F, Korali N (1999). Ultrasonographic diagnosis of hepatic fibrosis or cirrhosis. *Journal of Hepatology*.

[11] Runyon BA, Hoefs JC, Morgan TR (1988). Ascitic fluid analysis in malignancy-related ascites. *Hepatology*.

[12] Marin D, Furlan A, Federle MP, Midiri M, Brancatelli G (2009). Imaging approach for evaluation of focal liver lesions. *Clinical Gastroenterology and Hepatology*.

[13] Reynolds TB, Geller HM, Kuzma OT, Redeker AG (1960). Spontaneous decrease in portal pressure with clinical improvement in cirrhosis. *The New England Journal of Medicine*.

[14] Amini M, Runyon BA (2010). Alcoholic hepatitis 2010: a clinician’s guide to diagnosis and therapy. *World Journal of Gastroenterology*.

[15] Veldt BJ, Lainé F, Guillygomarc’h A (2002). Indication of liver transplantation in severe alcoholic liver cirrhosis: quantitative evaluation and optimal timing. *Journal of Hepatology*.

[16] Addolorato G, Leggio L, Ferrulli A (2007). Effectiveness and safety of baclofen for maintenance of alcohol abstinence in alcohol-dependent patients with liver cirrhosis: randomised, double-blind controlled study. *The Lancet*.

[17] Avanesyan A, Runyon BA (2010). Utilization of baclofen in maintenance of alcohol abstinence in patients with alcoholic hepatitis in a real-life setting. *Hepatology*.

[18] Hu KQ, Kyulo NL, Esrailian E (2004). Overweight and obesity, hepatic steatosis, and progression of chronic hepatitis C: a retrospective study on a large cohort of patients in the United States. *Journal of Hepatology*.

[19] Runyon BA (2009). Management of adult patients with ascites due to cirrhosis: an update. *Hepatology*.

[20] Haq SM, Dayal HH (2005). Chronic liver disease and consumption of raw oysters: a potentially lethal combination—a review of Vibrio vulnificus septicemia. *The American Journal of Gastroenterology*.

[21] Nair S (2010). Vitamin D deficiency and liver disease. *Gastroenterology and Hepatology*.

[22] Petta S, Camma C, Scazzone C (2010). Low vitamin d serum level is related to severe fibrosis and low responsiveness to interferon-based therapy in genotype 1 chronic hepatitis C. *Hepatology*.

[23] Spahr L, Coeythaux A, Giostra E (2005). Sleep alterations, hepatic encephalopathy and the histamine H1 receptor blocker hydroxyzine in patients with cirrhosis: a double blind RCT. *Hepatology*.

[24] Lee F-Y, Lee S-D, Tsai Y-T (1991). A randomized controlled trial of quinidine in the treatment of cirrhotic patients with muscle cramps. *Journal of Hepatology*.

[25] Mayo MJ, Handem I, Saldana S, Jacobe H, Getachew Y, Rush AJ (2007). Sertraline as a first-line treatment for cholestatic pruritus. *Hepatology*.

[26] Plank LD, Gane EJ, Peng S (2008). Nocturnal nutritional supplementation improves total body protein status of patients with liver cirrhosis: a randomized 12-month trial. *Hepatology*.

[27] Radvan GH, Vidikan P (1982). Meralgia paresthetica and liver disease. *Annals of Internal Medicine*.

[28] Garcia-Tsao G, Sanyal AJ, Grace ND (2007). Prevention and management of gastroesophageal varices and variceal hemorrhage in cirrhosis. *Hepatology*.

[29] Bruix J, Sherman M (2005). Management of hepatocellular carcinoma. *Hepatology*.

[30] Kuehn BM (2010). Alarming nonfatal overdose rates found for opioids, sedatives, and tranquilizers. *Journal of the American Medical Association*.

[31] de Lédinghen V, Mannant P-R, Foucher J (1996). Non-steroidal anti-inflammatory drugs and variceal bleeding: a case-control study. *Journal of Hepatology*.

[32] Rumack BH (2002). Acetaminophen hepatotoxicity: the first 35 years. *Journal of Toxicology—Clinical Toxicology*.

[33] Reuben A, Koch DG, Lee WM (2010). Drug-induced acute liver failure: results of a U.S. multicenter, prospective study. *Hepatology*.

[34] Doberneck RC, Sterling WA, Allison DC (1983). Morbidity and mortality after operation in nonbleeding cirrhotic patients. *The American Journal of Surgery*.

[35] Suman A, Barnes DS, Zein NN, Levinthal GN, Connor JT, Carey WD (2004). Predicting outcome after cardiac surgery in patients with cirrhosis: a comparison of Child-Pugh and MELD scores. *Clinical Gastroenterology and Hepatology*.

